# The Impact of Hyperthermia on Receptor-Mediated Interleukin-6 Regulation in Mouse Skeletal Muscle

**DOI:** 10.1371/journal.pone.0148927

**Published:** 2016-02-12

**Authors:** Steven S. Welc, Deborah A. Morse, Alex J. Mattingly, Orlando Laitano, Michelle A. King, Thomas L. Clanton

**Affiliations:** 1 University of Florida, Department of Applied Physiology & Kinesiology, College of Health and Human Performance, Gainesville, FL, United States of America; 2 Federal University of Vale do São Francisco, Physical Education School, Petrolina, Brazil; Tohoku University, JAPAN

## Abstract

In inflammatory cells, hyperthermia inhibits lipopolysaccharide (LPS)-induced interleukin-6 (IL-6) gene expression and protein secretion. Since hyperthermia alone stimulates IL-6 in skeletal muscle, we hypothesized that it would amplify responses to other receptor-mediated stimuli. IL-6 regulation was tested in C2C12 myotubes and in soleus during treatment with epinephrine (EPI) or LPS. In EPI-treated myotubes (100 ng/ml), 1 h exposure at 40.5°C-42°C transiently increased IL-6 mRNA compared to EPI treatment alone at 37°C. In LPS-treated myotubes (1 μg/ml), exposure to 41°C-42°C also increased IL-6 mRNA. In isolated mouse soleus, similar amplifications of IL-6 gene expression were observed in 41°C, during both low (1 ng/ml) and high dose (100 ng/ml) EPI, but only in high dose LPS (1 μg/ml). In myotubes, heat increased IL-6 secretion during EPI exposure but had no effect or inhibited secretion with LPS. In soleus there were no effects of heat on IL-6 secretion during either EPI or LPS treatment. Mechanisms for the effects of heat on IL-6 mRNA were explored using a luciferase-reporter in C2C12 myotubes. Overexpression of heat shock factor-1 (HSF-1) had no impact on IL-6 promoter activity during EPI stimulation, but elevated IL-6 promoter activity during LPS stimulation. In contrast, when the activator protein-1 (AP-1) element was mutated, responses to both LPS and EPI were suppressed in heat. Using siRNA against activating transcription factor-3 (ATF-3), a heat-stress-induced inhibitor of IL-6, no ATF-3-dependent effects were observed. The results demonstrate that, unlike inflammatory cells, hyperthermia in muscle fibers amplifies IL-6 gene expression to EPI and LPS. The effect appears to reflect differential engagement of HSF-1 and AP-1 sensitive elements on the IL-6 gene, with no evidence for involvement of ATF-3. The functional significance of increased IL-6 mRNA expression during heat may serve to overcome the well-known suppression of protein synthetic pathways occurring during heat shock.

## Introduction

Skeletal muscle produces IL-6 and other cytokines in response to receptor-mediated signals and from disturbances in internal homeostasis that result in cellular stress (reviewed in [1]). Receptor-mediated signal transduction occurs via abundant toll-like receptors (TLRs) [2,3], α- and β-adrenergic receptors [4,5], ATP/adenosine receptors [3,6], and TNFα and IL-1β receptors [7] present on muscle fibers. Ligands for these receptors are often present in the circulation in a variety of physiological and pathologic conditions, including disorders that can result in multiple organ dysfunction syndrome (MODS) [8], local muscle injury [9,10], heavy endurance exercise [11,12] and in sepsis [13,14]. IL-6 can also be released in response to intracellular stress following fatiguing exercise [15], hyperthermia [16,17], oxidative stress [18], glycogen depletion [19], accumulation of damaged proteins [20], and activation of the unfolded protein response of the endoplasmic reticulum [20].

In intact organisms, it would be rare that one IL-6 stimulus presents itself to muscle fibers in isolation. For example, in heavy endurance exercise, circulating levels of catecholamines [21], endotoxin [22] or damage-associated molecular patterns (DAMPs) [11,12] have often been reported, and there are simultaneous elevations in muscle temperature. At rest, human skeletal muscle is ≈2–4°C below core temperature [23], but in endurance exercise in hot environments it can rise to 0.5–1°C above core temperature [24], reaching values of 41°C in humans [24] and higher (44°C) in rodents [25]. The influence of temperature on the IL-6 response in exercise is illustrated by the observation that when whole body cooling suppresses natural elevations in core and muscle temperature in exercise, there are no apparent elevations in circulating IL-6 [26]. Therefore, using exercise as an example, there is evidence of significant interactions between temperature and other signaling pathways important for IL-6 regulation. These interactions may have additional importance in fever (core temperature: 38–41°C) or in heat illness (>40°C).

In this study, we evaluated the impact of hyperthermia, at various temperatures, on the responses to receptor-mediated IL-6 stimulation in skeletal muscle, using EPI and LPS as representative ligands. These have both previously been shown to independently have potent influences on IL-6 secretion in cultured myoblasts and in the whole organism [5,27,28]. We hypothesized that moderate elevations in temperature, within ranges characteristic of fever or in muscle during heavy exercise, would intensify the skeletal muscle responses to these stimuli. This idea was based on work by Inouye *et al*. [29], who showed that in the presence of HSF-1 (the principal mediator of the heat shock response) the chromatin structure of the human IL-6 promoter opens, making it more sensitive to both gene activator and repressor proteins. How this reveals itself depends on the cell type, the stimulus, and probably the activity of the particular activator or repressor proteins predominant at a given time and condition. Previous studies in inflammatory cells and fibroblasts have shown that hyperthermia inhibits LPS-induced IL-6 secretion [30,31]; whereas in gastrointestinal enterocytes, hyperthermia amplifies IL-1β-induced IL-6 [32,33]. How temperature influences IL-6 regulation in response to receptor-mediated stimuli in skeletal muscle is unknown.

Another aim of this study was to investigate the primary signaling pathways that support interactions between hyperthermia and receptor-mediated IL-6 expression in muscle. In previous work, we found two pathways particularly powerful in the response of the IL-6 gene to heat, HSF-1 induction and factors that stimulate activator protein-1 (AP-1) response element on the DNA promoter [20]. Using an IL-6 gene reporter system and HSF-1 mutant plasmids, we evaluate how temperature-related factors contribute to interactions with receptor-mediated stimuli. We hypothesize that HSF-1 is responsible for these interactions. Furthermore, we tested if the AP-1 response element mediates the EPI- or LPS-induced signals or if it assists in the potentiating effect of heat. A related line of inquiry determined if these experimental conditions altered expression of activating transcription factor-3 (ATF-3) in muscle. ATF-3 is a stress activated protein that inhibits LPS-induced IL-6 in fibroblasts and macrophages after hyperthermia [31]. Our results demonstrate that in skeletal muscle fibers, hyperthermia amplifies the mRNA responses to both EPI- and LPS-stimulation, despite induction of ATF-3 mRNA. In contrast, we found profound differences in the effects of hyperthermia on IL-6 protein secretion during EPI and LPS stimulation.

## Materials and Methods

### Chemicals and reagents

The following chemicals and reagents were used: Dulbecco’s Modification of Eagle’s Medium (DMEM) (Mediatech Inc, Mannassas, VA), lipopolysaccharide (LPS) derived from Escherichia coli 026:B6 and L-epinephrine (Sigma Chemical, St. Louis, MO), Isol-RNA Lysis reagent (5-Prime Inc, Gaithersburg, MD), dimethyl sulfoxide (DMSO) (Acros Organics, Fair Lawn, NJ), Dulbecco’s phosphate buffered saline (DPBS), standard fetal bovine serum (FBS) (HyClone, Logan, UT), horse serum (Lonza, Walkersville, MD), tissue protein extraction reagent (T-PER), protease inhibitor cocktail (Biotool), Verso cDNA synthesis kit (Thermo Scientific Inc, Rockford, IL), Taqman advanced fast master mix, Opti-MEM, Lipofectamine 2000 transfection reagent (Life Technologies, Carlsbad, CA), FuGENE HD transfection reagent, dual-luciferase reporter assay system (Promega Corp, Madison, WI), Bio-Rad protein assay (Bio-Rad Laboratories, Irvine, CA), BD OptEIA Mouse IL-6 ELISA kit (BD Biosciences, San Jose, CA). Accession numbers for relevant proteins and mRN are as follows: IL-6 (AAI38767.1); heat shock factor 1 (HSF-1) (AA960185); IL-6 mRNA (NM_031168) and activating transcription factor-3, ATF-3 mRNA (NM_007498).

### Cell culture

The C2C12 mouse myoblast cell line was purchased from the American Type Culture Collection (Mannassas, VA) and cultured in a water-jacketed humidified incubator set at 37°C in 5% CO_2_-95% atmospheric air (NAPCO 8000WJ, Thermo Scientific Inc, Marietta, OH). Laboratory cell stocks, passages 3–10, were routinely tested for mycoplasma infection using the method described by Zakharova et al. [34]. Cells were uniformly seeded to 6-well or 24-well cell culture plates (Corning Inc, Corning, NY). Cultures were grown in DMEM containing 4.5 g/L of glucose, L-glutamine and sodium pyruvate with 10% FBS. Once cells reached near confluence, differentiation was induced by switching to fresh differentiation medium (DMEM plus 2% horse serum) and differentiated for 5 days into multi-nucleated fibers (myotubes). Media was routinely exchanged every 1–2 days.

### Plasmids and reporter gene assays

Expression plasmids for the constitutively active (c.a.) HSF-1 mutant and dominant-negative (d.n.) HSF-1 mutant were obtained from Dr. Richard Voellmy (University of Florida, Gainesville, FL) and have previously been used and described [35–37]. The c.a. HSF-1 cDNA contains a deletion in the regulatory domain (amino acids 203–315) that is important to the transcriptional activity of HSF-1. The d.n. HSF-1 cDNA contains a deletion in the transactivation domain (amino acids 454–522) and can therefore bind DNA in target genes but not transactivate. This blocks target gene activation by endogenous HSF-1. The wild type (WT) mouse IL-6 promoter/luciferase reporter containing all regulatory elements (~1277 nucleotides) (mIL-6.Luc) and mouse IL-6 promoter/luciferase reporter gene plasmid with a mutation in the putative AP-1 binding site (mIL-6.Luc AP-1) were a gift from Dr. Gail Bishop (University of Iowa, Iowa City, IA) and have previously been used and described [38]. Plasmid DNA was amplified and isolated from bacterial cultures using Endotoxin-Free Maxi Prep Kits (Qiagen, Valencia, CA), precipitated in ethanol and resuspended in Tris-EDTA (TE) buffer for transfections in culture. All cultures used for reporter assays were co-transfected with pRL-TK (*Renilla*) (control reporter vector), mIL-6.Luc or mIL-6.Luc AP-1, and c.a.HSF-1 or d.n. HSF-1 or their empty vector (EV) as labeled.

Myoblasts were transfected with plasmid DNA at 60–80% confluence using FuGENE HD Transfection Reagent at a 3.5:1 ratio of reagent to total DNA. Sixteen hours following transfection, muscle cells were differentiated into myotubes by incubation in differentiation medium. Following differentiation into myotubes, transfection efficiency was approximately 25%. For all studies, 5-day differentiated myotubes were treated as described in cellular experiments (below) in differentiation media for 6 h. For reporter experiments, cells were harvested in 500 μl of passive lysis buffer (PLB), and luciferase activity was determined by normalizing firefly luciferase activity to *Renilla* luciferase activity using a Dual-Luciferase Reporter Assay. Luciferase activity of cell lysates was measured in relative light units (RLU) (Biotek Synergy 2 platform, Winooski, VT) and normalized (normalized RLU = RLU firefly luciferase/RLU Renilla luciferase).

### RNA interference

C2C12 myoblasts were transiently transfected in six-well plates with small interfering RNA (siRNA) sequences for ATF-3. Two validated siRNA sequences were evaluated for knockdown efficacy (Life Technologies, Carlsbad, CA). RNA oligos were transfected into myoblasts using Lipofectamine 2000, according to the manufacturer’s protocol. Briefly, ATF-3 siRNA and Lipofectamine 2000 were diluted separately in Opti-MEM. The diluted Lipofectamine 2000 reagent was incubated for 5 min and was then added to the siRNA mixture (500 μl/well). The lipid/siRNA mixtures were allowed to complex for 20 min during which the cells were rinsed with Opti-MEM and incubated in a final volume of 2 ml Opti-MEM/well. Lipofectamine/siRNA complexes were then applied to the cells (final transfection volume 2.5 ml/well). Cultures were incubated with the transfection mixture overnight. Parallel cultures were transfected with a nonsilencing control scramble (CON) to control for the effects of siRNA delivery. The next morning, transfection medium was removed and replaced with growth medium. Cultures were maintained for 24 h before experimental treatment.

### Cellular experiments

A variety of experiments were conducted to evaluate C2C12 cell responses to varying stress stimuli: heat shock (40.5–42°C), HSF-1 overexpression, HSF-1 inhibition and pharmacological intervention (EPI, LPS). All results were obtained from four to five separate experiments, two samples per experiment, to ensure reproducibility.

### Heat treatment

Myotubes were supplemented with EPI (100 ng/mL) or LPS (1 μg/mL) and maintained at 37°C or acutely treated in a second water-jacketed, humidified CO_2_ incubator pre-set to an environmental temperature (T_ENV_) of 40.5, 41, or 42°C (Forma Scientific 3154, Marietta, OH). T_ENV_ within the incubator was monitored using an YSI thermistor, accurate to 0.01°C. Once cells were placed in the incubator it took ≈20 min for the T_ENV_ to reach the desired temperature. Treated cells were either maintained at 37°C for the entire experiment (6 h) or exposed to an experimental temperature (40.5, 41, or 42°C) for the first hour and 37°C for the remaining time (0, 1, 2, 5 or 11 h). Cells were harvested following the culmination of heat treatment (1 h) or after heat and recovery; unheated cells were matched and kept at 37°C for the entirety of the experiment. A lactate dehydrogenase (LDH) activity assay was performed to measure cell viability of cultures treated with EPI or LPS at basal and under heated conditions. There were no significant changes in LDH activity between treatments [data not shown, EPI (P = 0.5812), LPS (P = 0.7360)]. Cellular supernatant was collected; cells were lysed in Isol-RNA Lysis reagent, PLB or T-PER and stored at -80°C immediately.

### *Ex Vivo* Muscle Preparation Experiments

Male 3–4 mo old C57BL6/J mice were purchased from Jackson Laboratories (Bar Harbor, ME) and housed at the University of Florida Animal Care Facilities. All animal procedures were approved by the University of Florida's Institutional Animal Care and Use Committee. Mice (n = 24) were anesthetized with isoflurane prior to muscle isolation. The soleus muscles were then rapidly excised and prepared at room temperature in oxygenated Krebs Ringer solution, containing (in mM): 121 NaCl, 5.9 KCl, 2.0 CaCl_2_, 1.0 MgCl_2_, 0.6 Na_2_HPO_4_, 21 NaHCO_3_, 0.45 Na_2_SO_4,_ 11.5 glucose and 10 μM D-tubocurarine. Baths were continuously bubbled with 95% O_2_/5% CO_2_, mounted, and placed in 2 ml tissue baths, preset to 35°C, a temperature based on direct measurements of muscle temperature in unanesthetized resting mouse limb muscle [39]. Optimal length of the muscle was adjusted to a standard preload of 1 g and the muscles were not electrically stimulated at any time in the protocol. Elevating bath temperature required ∼5 min. Two sets of experiments were performed, a “high dose” and a “low dose” treatment. The high dose experiments mimicked concentrations used in the C2C12 studies described above, i.e. EPI = 100 ng/ml; LPS = 1 μg/ml. One soleus from each mouse was maintained at 35°C for 2 h (sham controls), while the other soleus was heated to 41°C for 1 h, followed by recovery at 35°C for 1 h. Sixty μL bath samples were collected at 1 and 2 h for Luminex multiplex analysis into a protease inhibitor cocktail, using manufacturer’s instructions. The results were compared against fresh physiological buffer used in the tissue baths.

A second series of “low dose” treatments were carried out identical to the high dose experiments, except that the concentrations of EPI and LPS were designed to resemble concentrations found *in vivo*. In all other respects the experiments were identical. The low dose EPI was 1 ng/ml, near plasma values measured in rats during passive heat exposure to 41°C, i.e. ≈0.7 ng/ml [40] and similar to values found in human plasma immediately at the end of exhaustive endurance exercise, i.e. ≈1.2 ng/ml [41]. The low dose LPS used was 200 pg/ml, a value in the mid-range found in plasma in humans during severe septic shock, i.e. ≈110–726 pg/ml [13]. Following completion of the protocol the muscles were quickly blotted on tissue paper, flash-frozen in liquid nitrogen, and stored at −80°C for subsequent mRNA analyses. RNA isolation and real-time RT-PCR analysis

Tissues and cells were lysed in ISOL-RNA Lysis Reagent according to the manufacturer’s instructions. Briefly, RNA was separated from protein and DNA by the addition of bromochloropropane and precipitation in isopropanol. After a 75% ethanol wash and resuspension in diethyl pyrocarbonate H_2_O, purity of RNA samples was quantified using spectrophotometry. Total mRNA (1 μg) was then reverse transcribed using Verso cDNA Synthesis Kit. Pre-formulated TaqMan Gene Expression Assays were purchased from Applied Biosystems for the following mouse genes: Interleukin-6 (IL-6) (Mm00446191_m1), Activating Transcription Factor -3 (ATF-3) (Mm00476032_m1), Glyceraldehyde-3-Phosphate (GAPDH) (Mm99999915_g1), Beta Actin (ACTB) (Mm00607939_s1), and Hypoxanthine Guanine Phosphoribosyl Transferase (HPRT) (Mm01545399_m1). Relative quantitative real time reverse-transcription polymerase chain reaction (RT-PCR) was performed using the TaqMan Fast Advanced Master Mix, and reactions were performed in duplicate using 96-well optical plates on a StepOnePlus Real-Time PCR System (Life Technologies). Candidates for housekeeping genes, GAPDH, HPRT, and ACTB, were tested for stability over various experimental treatments as previously shown [17]. GAPDH was used as the endogenous control to normalize the samples. GAPDH has previously been used as a stable housekeeping gene for C2C12 cells treated with EPI and LPS [5,42] and in heated cells [17]. Data demonstrating its stability in conditions of this study can be found in, see supporting information, S1 Fig. The changes in IL-6 and ATF-3 were independent of changes in the level of mRNA for GAPDH. Relative quantitation was done using the ΔΔCT method, where CT is the cycle threshold, and all untreated samples were normalized to 1. For soleus experiments, total RNA was extracted from homogenized soleus muscles using TRIzol^®^ reagent (Invitrogen) following the manufacturer's instructions. Total mRNA (1 μg) was then reverse transcribed using Verso cDNA Synthesis Kit using the primer sets as described above.

### Protein extraction and quantification

Cells were washed with DPBS without calcium or magnesium and lysed for 15–20 mins at 4°C by the addition of 350 μl of T-PER lysis buffer containing a protease inhibitor cocktail. The lysate was transferred to clean microcentrifuge tubes and stored at -80°C for subsequent analysis. Total protein was quantified using the Bio-Rad protein assay using bovine gamma globulin (IgG) as a standard. The lysis buffer was tested for possible interference with protein quantification and ELISA assays through the generation of standard curves with varying concentrations of T-PER lysis buffer. The concentration 0.1X T-PER was determined to be compatible with the Bio-Rad protein assay.

### Protein measurements using ELISA and Luminex

A mouse BD OptEIA IL-6 ELISA kit was used to evaluate IL-6 protein. Briefly, 96-well clear flat bottom non-treated polystyrene microtest plates (BD Falcon, Franklin Lakes, NJ) were incubated with anti-mouse IL-6 capture antibody overnight at 4°C. The next day, plates were washed and blocked with 200 μl of assay diluent (PBS + 10% heat-inactivated FBS) for 1 h. Frozen samples of cellular supernatant were brought to room temperature, diluted (if necessary), and samples were plated. The working detector was prepared by adding Streptavidin-Horse Radish Peroxidase (HRP) conjugate to the biotinylated anti-mouse IL-6 antibody. The working detector binds to the IL-6 captured by the plate coated antibody. A substrate solution, reactive with HRP, is then added to the wells and a colorimetric product was formed in proportion to the amount of mouse IL-6 present. Concentrations of samples in pg/ml were interpolated using a 5-parameter logistic standard curve.

For measures of protein secreted into the muscle baths from soleus muscles a Luminex system was utilized for IL-6 and other proteins, using a MILLIPLEX MAP Mouse Immunology 25-Plex or 32 PLEX Assays (Millipore, Billerica, MA) as previously described [17].

### Statistics

All data was analyzed using SAS JMP Pro 10 software. Values of central tendency were expressed as means ± SEM when sample populations were parametric and means compared. Cellular experiments were repeated 4–5 separate times with 2 samples taken from each experiment (total n = 8–10). Data were analyzed by t test and multi-way analysis of variance (ANOVA) where appropriate; post hoc analysis was performed by comparing individual means using mean contrasts.

The mRNA responses measured in soleus muscles were normalized to a naïve control soleus muscle and expressed as a fold change. The samples were paired between heated and unheated solei from the same mouse. Tests of significant effects of heat were determined using a Wilcoxon signed ranks for paired measurements. Measurement of soleus IL-6 secretion using the Luminex assay were nonparametric and were tested for the effects of heat exposure in paired solei using the Wilcoxon signed ranks test for paired samples. To test for significant elevations of IL-6 in the bath, Wilcoxon signed ranks for unpaired measurements was used. The hypothesis tested was whether the IL-6 was > the threshold for detection of IL-6 using the Luminex assay, the same as the values for fresh buffer.

## Results

### Hyperthermia potentiates EPI- and LPS-induced IL-6 gene expression and promoter activity in C2C12 myotubes

**Fig 1:**
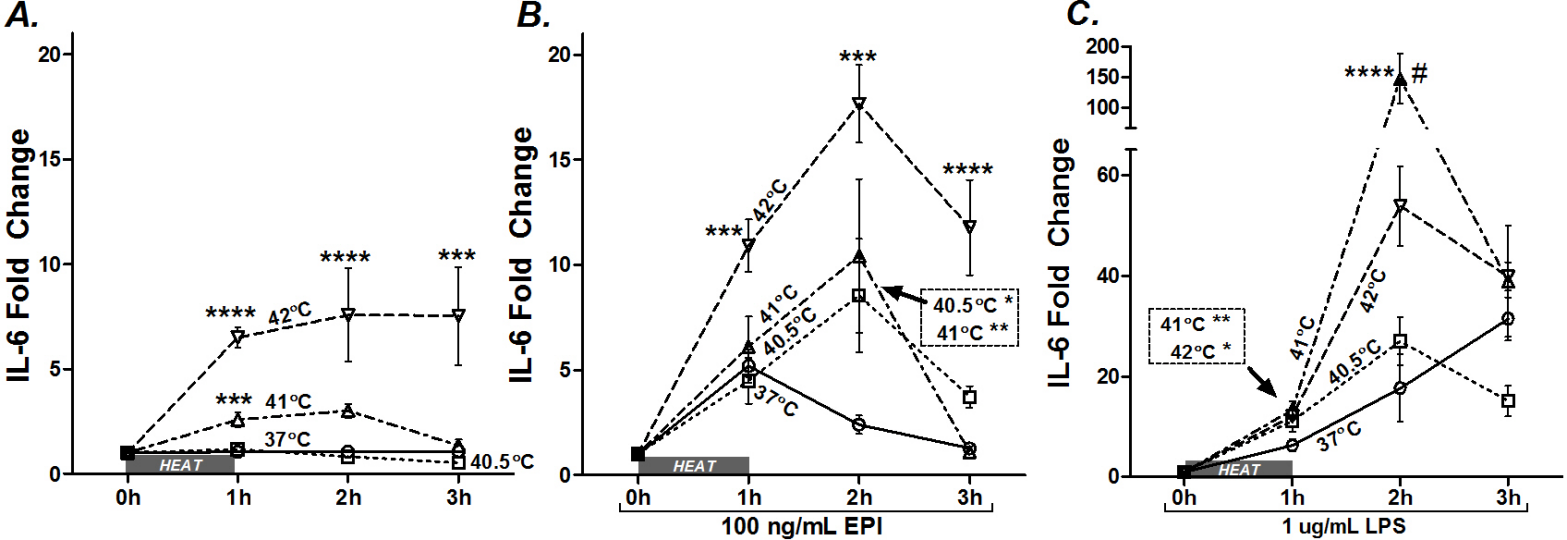
Hyperthermia potentiates EPI and LPS-induced IL-6 mRNA. **A)** myotubes were maintained at 37°C (○) or exposed to 40.5 (□), 41 (Δ) or 42°C (▽) for 1 h (1 h) and harvested or allowed to recover at 37°C for 1 (2 h) or 2 h (3 h total). Myotubes were treated with **B)** EPI (100 ng/ml) or **C)** LPS (1 μg/ml) and maintained at 37°C or exposed to hyperthermia for 1 h and then immediately harvested or allowed to recover at 37°C for 1 (2 h) or 2 h (3 h). Results from multiple cultures in 4–5 independent experiments, two samples per experiment. Multi-way ANOVA results for testing the effects of temperature within each treatment group A) heat (P = 0.0007), B) EPI (P<0.0001), and C) LPS (P = 0.0013). Post-ANOVA least squares contrasts was used between means at specific time points. * Comparison vs. unheated sample; ^#^ comparison vs. 41°C treated samples; * or ^#^ (P<0.05), ** (P<0.01), *** (P<0.001), and **** (P<0.0001).

A series of side-by-side experiments were designed to test the interaction of varying intensities of heat (40.5, 41, and 42°C) with EPI- or LPS-stimulated IL-6 gene expression. For this series, we treated myotubes with supaphysiologic concentrations of EPI (100 ng/ml) or LPS (1 μg/ml) and immediately exposed them to 37, 40.5, 41, or 42°C for 1 h. Cells were harvested or returned to 37°C for 1 or 2 h of recovery before being harvested. In the heat alone group, IL-6 mRNA expression was consistently amplified as a function of temperature ≥41°C (Fig 1A), confirming our previous study [17]. Similarly, myotubes treated with heat (40.5, 41, or 42°C) potentiated EPI-stimulated IL-6 mRNA (Fig 1B). This effect was relatively preserved across all 3 hours of evaluation (Fig 1B). Note that at 1 h after heat (2 h time point), in the presence of EPI, IL-6 mRNA was elevated ≈2 fold at 40.5°C compared to EPI alone, a temperature well in the range of exercising muscle and fever, but one that does not, by itself, stimulate IL-6 mRNA (Fig 1A). IL-6 mRNA was also augmented in LPS-treated myotubes following hyperthermia (Fig 1C). Interestingly, treatment with the intermediate temperature (41°C) resulted in the greatest amplification (≈7 fold above the response of LPS alone), showing maximal potentiation at 1 h of 37°C recovery (2 h time point).

**Fig 2:**
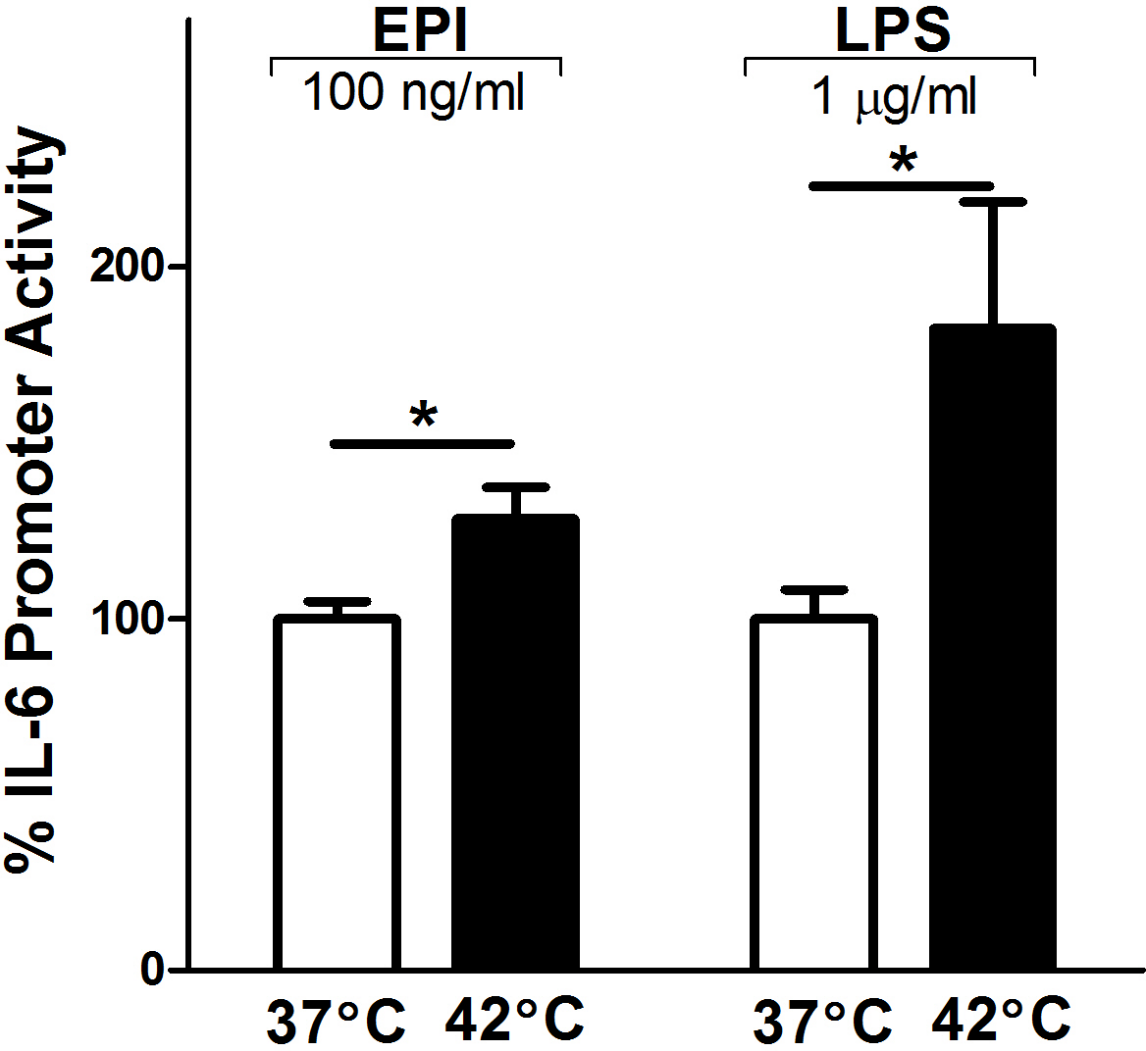
Hyperthermia potentiates IL-6 promoter activity. Activation of mouse IL-6 promoter/luciferase gene by heat shock. C2C12 myoblasts were transfected with wild-type full-length mouse IL-6 promoter/luciferase (mIL-6.Luc) reporter gene plasmid plus pRL-TK (*Renilla*) plasmid overnight, then differentiated over 5 days into myotubes. On day 5, myotubes were treated with EPI (100 ng/ml) or LPS (1 μg/ml) at 37°C for 6 h or at 42°C for 1 h with a recovery period at 37°C for 5 h. Luciferase activity in cell lysates was measured and normalized [i.e. normalized to relative light units (RLU) = RLU firefly luciferase/RLU *Renilla* luciferase]. Luciferase activity of the myotubes maintained at 37°C was set at 100% activity. Results from multiple cultures, five independent experiments, two samples per experiment; ANOVA, * Comparison during matching experimental treatments 37 vs. 42°C; P<0.05.

In a separate experiment, we tested whether heat shock induces increased IL-6 gene activation, using a luciferase reporter gene plasmid, as previously described [20]. Myoblasts were treated with EPI, LPS and/or subjected to heat shock and recovery (42°C 1 h + 37°C 5 h) or to matched control conditions (37°C for 6 h). The results are shown in Fig 2, where the luciferase activity (expressed in relative light units (RLUs)) of cell lysates were compared from EPI- and LPS- treated myotubes that were heated or left at control temperature. For clarity, the data are set to 100% of activity during EPI or LPS stimulation, without hyperthermia. Hyperthermia potentiated mIL-6.Luc promoter activity (Fig 2) in both EPI- (≈128% activity, P<0.05) and LPS-treated (≈182% activity, P<0.05) myotubes in a manner similar to the IL-6 mRNA responses described in Fig 1. Together these results demonstrate that heat potentiates EPI- and LPS-induced IL-6 gene expression.

### HSF-1 differentially regulates EPI and LPS-stimulated IL-6 promoter activity

**Fig 3:**
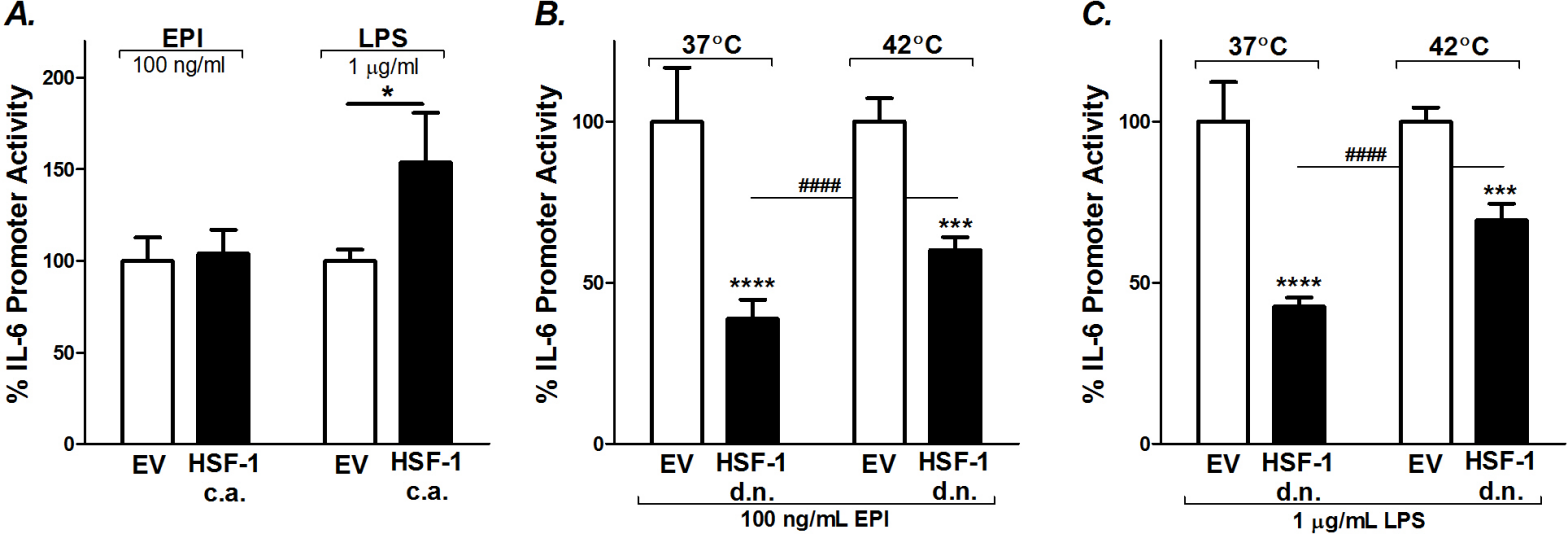
Effects of HSF-1 on EPI- and LPS-induced IL-6 promoter activity. A) C2C12 myoblasts were transfected with mIL-6.Luc, pRL-TK and constitutively active (c.a.) HSF-1 or its empty vector (EV) and differentiated into myotubes. Myotubes were treated with EPI (100 ng/ml) or LPS (1 μg/ml) at 37°C 6 h. B and C) The effects of HSF-1 inhibition on EPI- and LPS-induced IL-6 promoter activity during basal (37°C 6 h) and heated (42°C 1 h and 37°C 5 h) conditions. In these experiments C2C12 myoblasts were transfected with mIL-6.Luc, pRL-TK together with a dominant negative (d.n.) HSF-1 mutant or its EV and differentiated into myotubes. Results represent multiple cultures, 5 independent experiments, two samples per experiment; multiway ANOVA testing. The effects of each treatment group at individual time points are from post-ANOVA least squares contrasts between means. * Comparisons vs. EV; ^#^ comparisons among d.n. HSF-1 myotubes at 37 vs. 42°C; * (P<0.05),*** (P<0.001), and **** or ^####^ (P<0.0001).

To determine if HSF-1, in the absence of heat, is sufficient to potentiate EPI- or LPS-induced IL-6 transcriptional activation, we overexpressed HSF-1 using (c.a.HSF-1) [20]. C2C12 myoblasts were co-transfected with the mIL-6.Luc reporter and c.a.HSF-1 or its empty vector (EV), then differentiated and treated with EPI or LPS for 6 h. At 37°C, HSF-1 overexpression significantly potentiated LPS-induced IL-6 promoter activity (≈154%, P<0.05), but this was not seen in the EPI-treated myotubes (Fig 3A). Therefore, the responses to HSF-1 overexpression are distinctly different during EPI- vs. LPS stimulation.

Next, we tested the effects of inhibiting HSF-1 on EPI- and LPS-stimulated IL-6 promoter activity (Fig 3B and 3C). C2C12 myoblasts were co-transfected with mIL-6.Luc and a dominant negative HSF-1 construct (d.n.HSF-1) or EV. Inhibition of HSF-1 attenuated the EPI-induced IL-6 promoter activity in myotubes during basal conditions (≈61%, P<0.0001) and in heated conditions (≈40%, P<0.001) (Fig 3B). Similarly, in LPS-treated cells, mIL-6.Luc reporter activation was reduced during basal (≈57%, P<0.0001) and heated (≈31%, P<0.001) conditions (Fig 3C). In combination with our previous results [20], these outcomes suggest that basal HSF-1 activity is critical for IL-6 signal induction.

### Heat-induced potentiation of EPI- and LPS-stimulated IL-6 promoter activity is mediated through its AP-1 regulatory element

**Fig 4:**
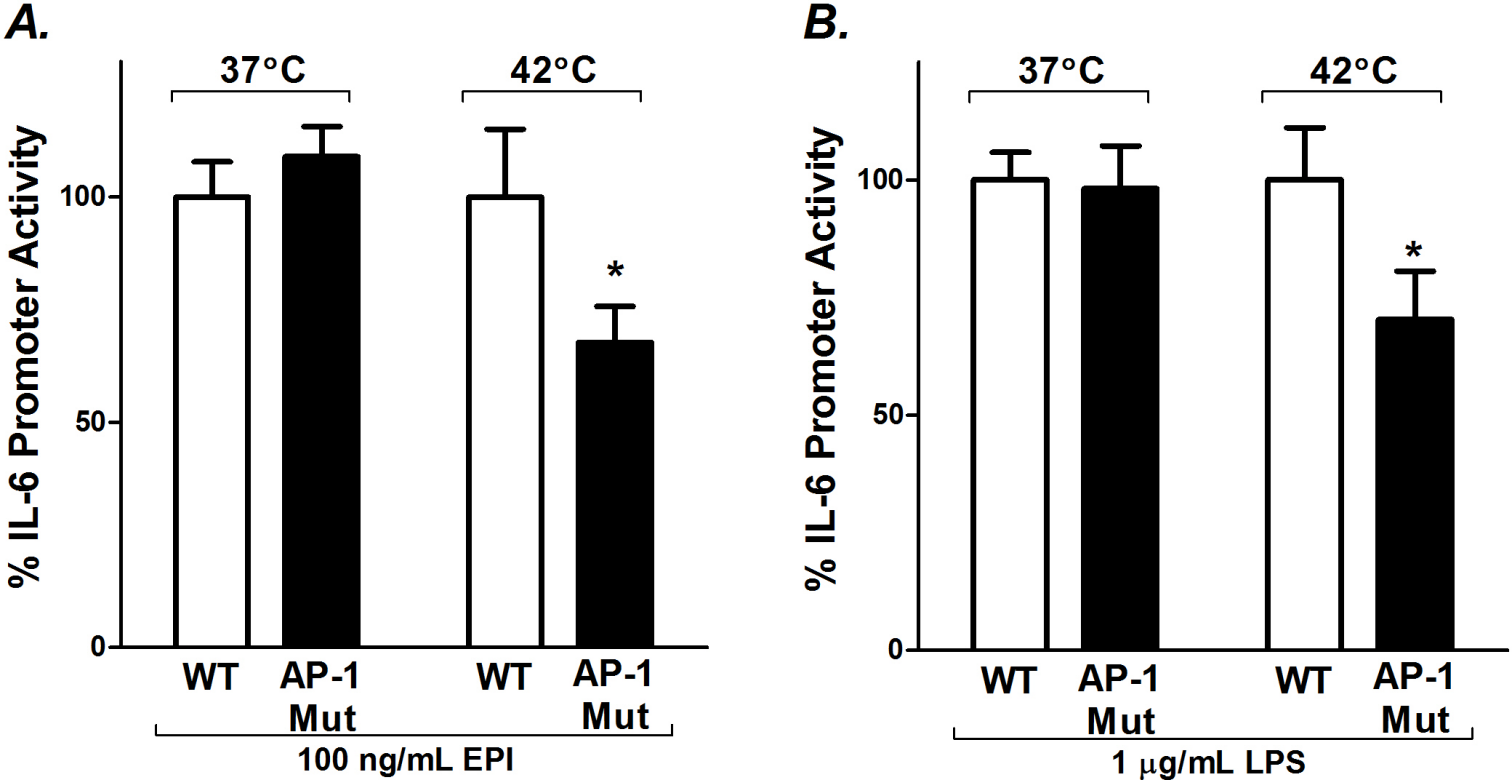
Mutation of the AP-1 binding site of the IL-6 promoter attenuates EPI and LPS stimulated IL-6 promoter activity under heated conditions. C2C12 myoblasts were transfected with the wild-type mIL-6.Luc (WT) or with a mutated AP-1 binding site (AP-1 Mut) plus the pRL-TK plasmid. Myotubes were grown and treated as described in Fig 2. The effects of AP-1 mutation on A) EPI- and B) LPS-induced IL-6 promoter activity during basal and heated conditions. Luciferase activity was measured (RLU), normalized, and calculated as a percentage of maximal promoter activity as described in Fig 2. Results from multiple cultures, five independent experiments, two samples per experiment; ANOVA, * Comparisons with EV under same experimental conditions; P<0.05.

In these experiments we tested whether the influence of hyperthermia on LPS or EPI stimulated myotubes is operating through the AP-1 response element [20]. It has previously been determined that pharmacological inhibitors of JNK and p38 MAPK, mediators upstream of AP-1, attenuate EPI- and LPS-induced IL-6 synthesis in myoblasts [5,42]. To test the involvement of AP-1, we used a mIL-6.Luc reporter with mutated AP-1 regulatory sites (mIL-6.Luc AP-1). Promoter activities were compared against the full-length wild-type promoter (mIL-6.Luc) in cultures treated with EPI and LPS under basal and hyperthermic conditions. During basal conditions (37°C), the AP-1 mutation had no effect on EPI-induced IL-6 promoter activity (Fig 4A). However, during heated conditions the mutation decreased EPI-induced IL-6 promoter activity by ≈32% (P<0.05) (Fig 4A). Similarly, the AP-1 mutation had no effect on LPS-induced IL-6 promoter activity during basal conditions and reduced the influence of heat on LPS-induced IL-6 promoter activity by ≈30% (P<0.05) (Fig 4B).

### Effects of hyperthermia on EPI- and LPS-induced IL-6 protein production

**Fig 5:**
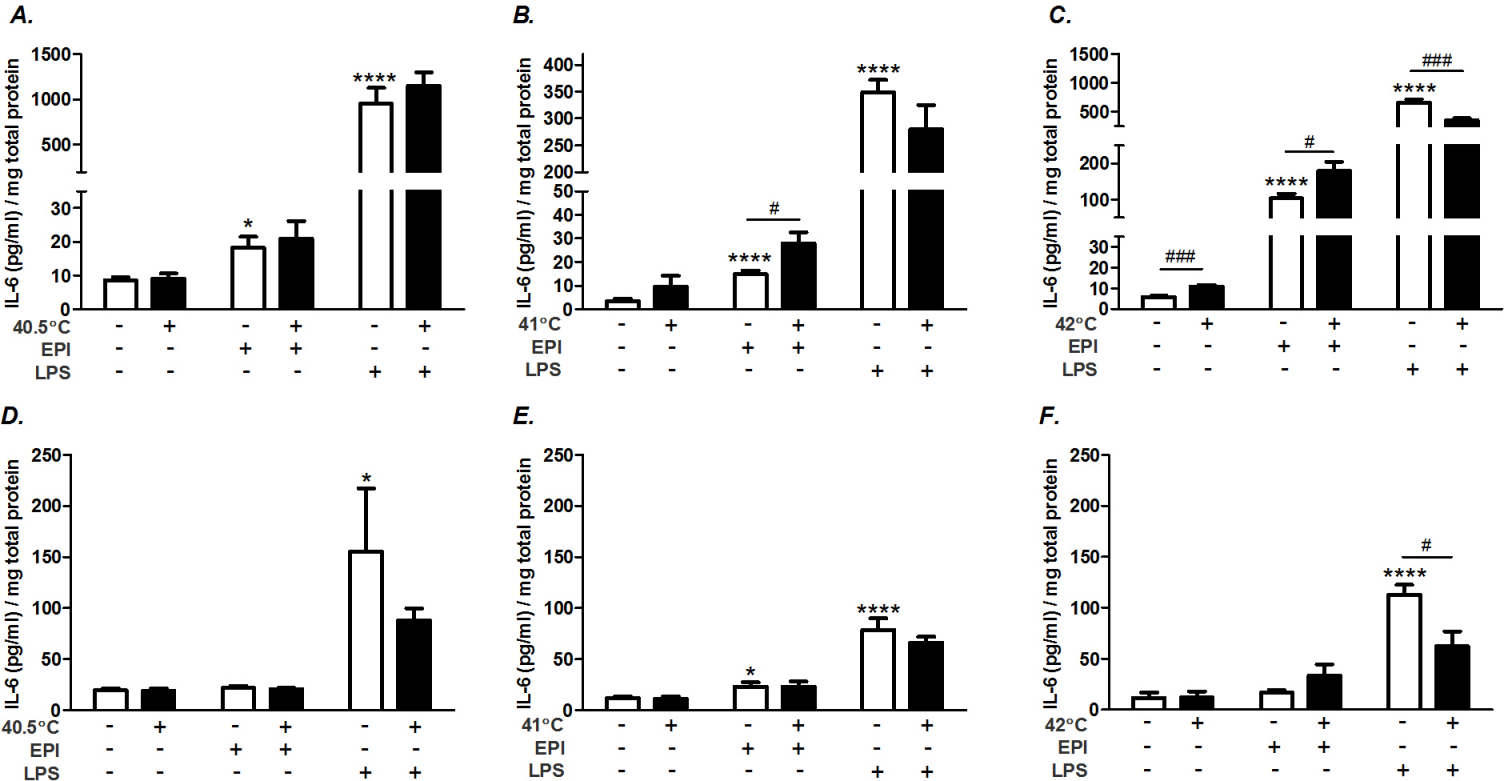
Hyperthermia differentially regulates IL-6 protein. Myotubes were maintained at 37°C or exposed to 40.5°C (A, D), 41°C (B, E) or 42°C (C,F) for 1 h and then maintained at 37°C for 5 h. Select cultures were co-stimulated with EPI, or LPS. Supernatants (A,B,C) and cell lysates (D,E,F) were sampled for IL-6 protein analysis by ELISA. Values are expressed relative to the total protein content from cells in each well. Results from multiple cultures, four independent experiments, two samples per experiment; ANOVA. * Comparison vs. 37°C control; ^#^ comparison during matching experimental treatments 37 vs. 42°C; * or ^#^ (P<0.05), *** or ^###^ (P<0.001), and **** (P<0.0001).

IL-6 protein was measured by ELISA in the supernatant and the myofiber lysates, 6 h after the onset of treatment (Fig 5). At temperatures of 41°C or 42°C, hyperthermia potentiated the levels of secreted IL-6 during EPI stimulation (Fig 5B and 5C). At this time point, IL-6 protein within the lysates was minimally affected by EPI, heat or EPI plus heat (Fig 5D–5F). In contrast, hyperthermia had no significant effect on LPS-induced IL-6 secretion at 40.5 and 41°C exposures (Fig 5A and 5B). However, at 42°C exposures, LPS-induced secretion was reduced compared to LPS alone (Fig 5C). This effect was also evident in the lysates at 42°C (Fig 5F), suggesting that this effect was related to synthesis and not just secretion. Therefore, the effects of hyperthermia on protein synthesis and secretion are uniquely different between EPI and LPS stimulation. Whereas during EPI stimulation hyperthermia potentiated secretion of IL-6, during LPS stimulation it either had no effect or inhibited secretion.

**Fig 6:**
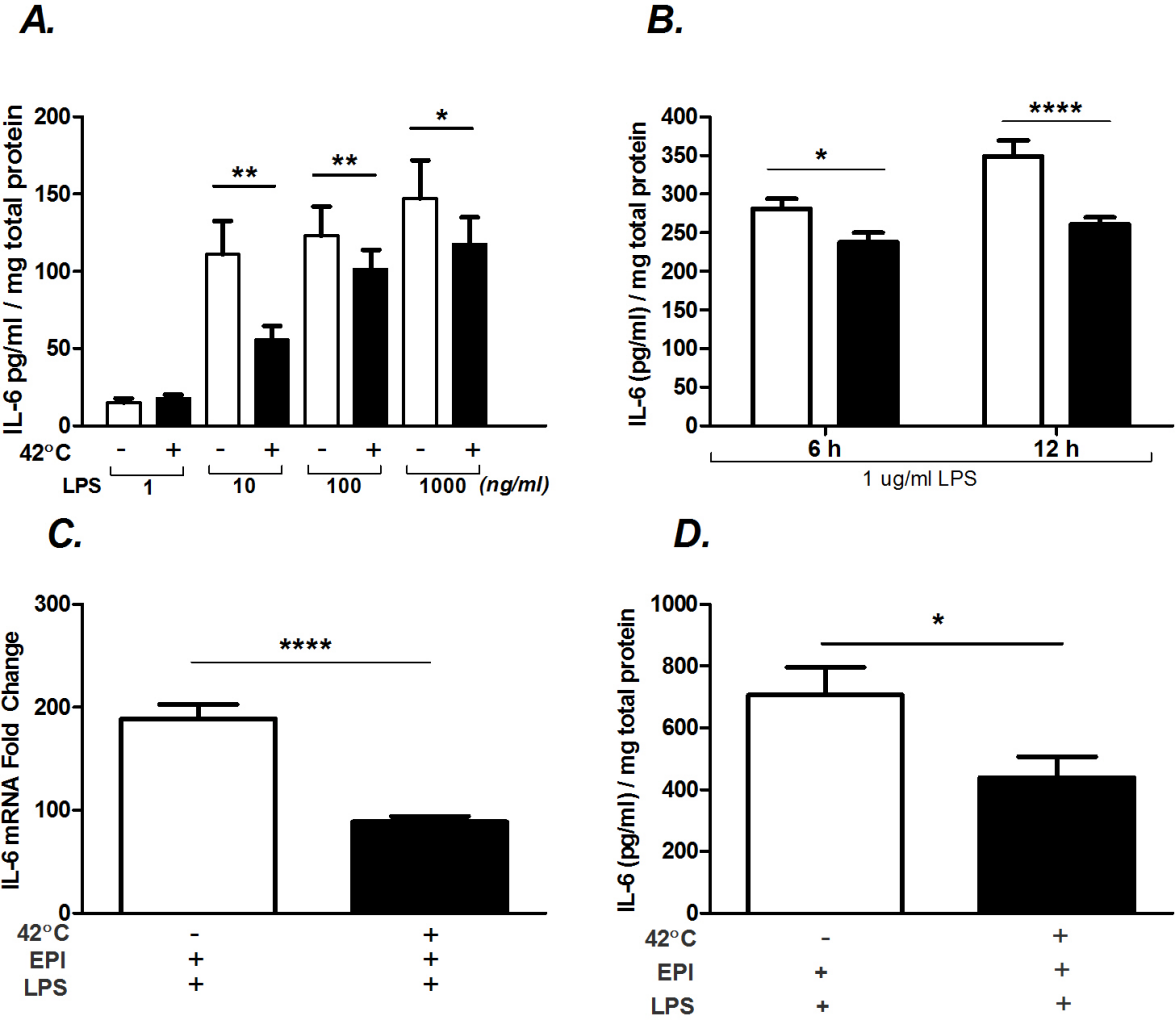
Hyperthermia attenuates LPS-induced IL-6 protein. A) Myotubes were treated with LPS (1–1000 ng/ml) and at 37°C 6 h or 42°C 1 h and 37°C 5 h. B) Myotubes were treated with LPS (1 μg/ml) and at 37°C 6 h or 42°C 1 h and 37°C 5 h. C and D) Myotubes were co-stimulated with LPS (1 μg/ml) plus EPI (100 ng/ml) and 37°C 6 h or 42°C 1 h and 37°C 5 h. Supernatants (A,B,D) were sampled for IL-6 protein analysis using ELISA. Values are expressed relative to the total protein content from cells in each well. C) IL-6 mRNA was evaluated by qRT-PCR. Results from multiple cultures, 4 independent experiments, 2 samples/experiment; ANOVA. * Comparison during matching experimental treatments 37 vs. 42°C; * (P<0.05), ** (P<0.01), and **** (P<0.0001).

The inhibitory influences of hyperthermia during LPS stimulation may lie in the “overstimulation” of myotubes. That is, transcriptional and translational machinery may not be able to further respond when operating at a maximum rate. To address this possibility, we tested whether the effects of hyperthermia on IL-6 protein secretion would be impacted by lowering the concentration of LPS-stimulation during heat. As shown in Fig 6A, there was a dose-dependent effect of LPS on IL-6 secretion, when tested over 2 orders of magnitude. Interestingly, the inhibitory effects of hyperthermia on IL-6 secretion were even more evident at low doses of LPS. This demonstrates that the inhibitory effect of hyperthermia on secretion during LPS exposure is present across multiple stimulation intensities. Note that no effects of LPS on IL-6 secretion could be seen at 1 ng/ml, which is above the level of LPS measured in plasma of patients with severe endotoxic shock [13].

Another potential explanation for why IL-6 protein is lower in heat during LPS co-stimulated myotubes at 6 h is that heat shock delays protein synthesis. To test for this, we measured secreted IL-6 protein in LPS-stimulated myotubes, with and without heat, over a longer time. Secreted IL-6 protein was also inhibited by heat when allowed to accumulate for 12 h after the onset of treatment (Fig 6B). Therefore, the effects of heat on LPS-induced IL-6 protein appear to be inhibitory and do not just reflect a delay in synthesis.

It was previously shown that co-stimulation of myoblasts with EPI and LPS synergistically increased IL-6 mRNA and protein synthesis [5]. We tested whether superimposing hyperthermia in addition to EPI + LPS stimulation would potentiate IL-6 mRNA and protein. The addition of heat shock attenuated the IL-6 mRNA response (Fig 6C) and the protein response to EPI + LPS co-stimulation (Fig 6D).

### Tests of underlying mechanisms

**Fig 7:**
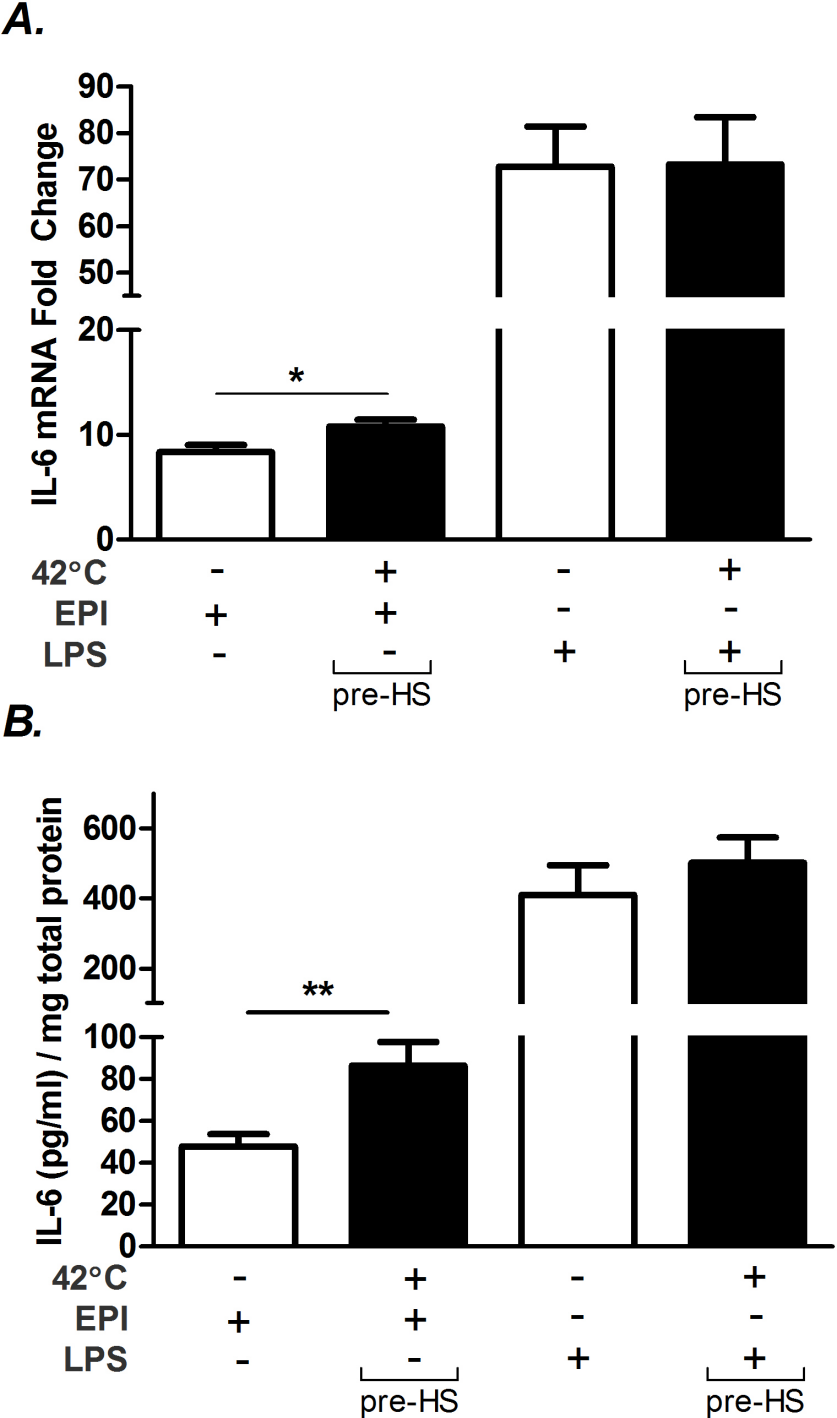
The timing of hyperthermia affects IL-6 regulation. Myotubes were kept at 37 or 42°C 1 h and then stimulated with EPI (100 ng/ml) or LPS (1 μg/ml) and returned to 37°C for 5 h. A) IL-6 mRNA was measured by qRT-PCR and B) IL-6 protein was measured by ELISA. Values are expressed relative to the total protein content from cells in each well. Results from multiple cultures, 4 independent experiments, 2 samples per experiment; ANOVA. * Comparison during matching experimental treatments 37 vs. 42°C; * (P<0.05), ** (P<0.01).

Our results differ in part from Takii et al. [31] who reported that hyperthermia consistently inhibited IL-6 mRNA and protein production during LPS stimulation. One potential explanation is a difference in methodology. In their experiments, Takii et al. [31] heated murine embryonic fibroblasts (MEFs) and peritoneal macrophages (MΦ) prior to exposure to LPS, as opposed to immediately combining LPS with heat exposure (Figs 1–5). Therefore, we repeated our experiments using a similar pre-heating protocol. EPI-induced IL-6 mRNA (Fig 7A, P<0.01) and protein (Fig 7B, P<0.05) responses were amplified, as before, at the 3 and 6 h time points, respectively. However, no effect of 42°C on either IL-6 mRNA or on protein was seen during LPS stimulation using the pre-heating protocol (Fig 7A and 7B).

**Fig 8:**
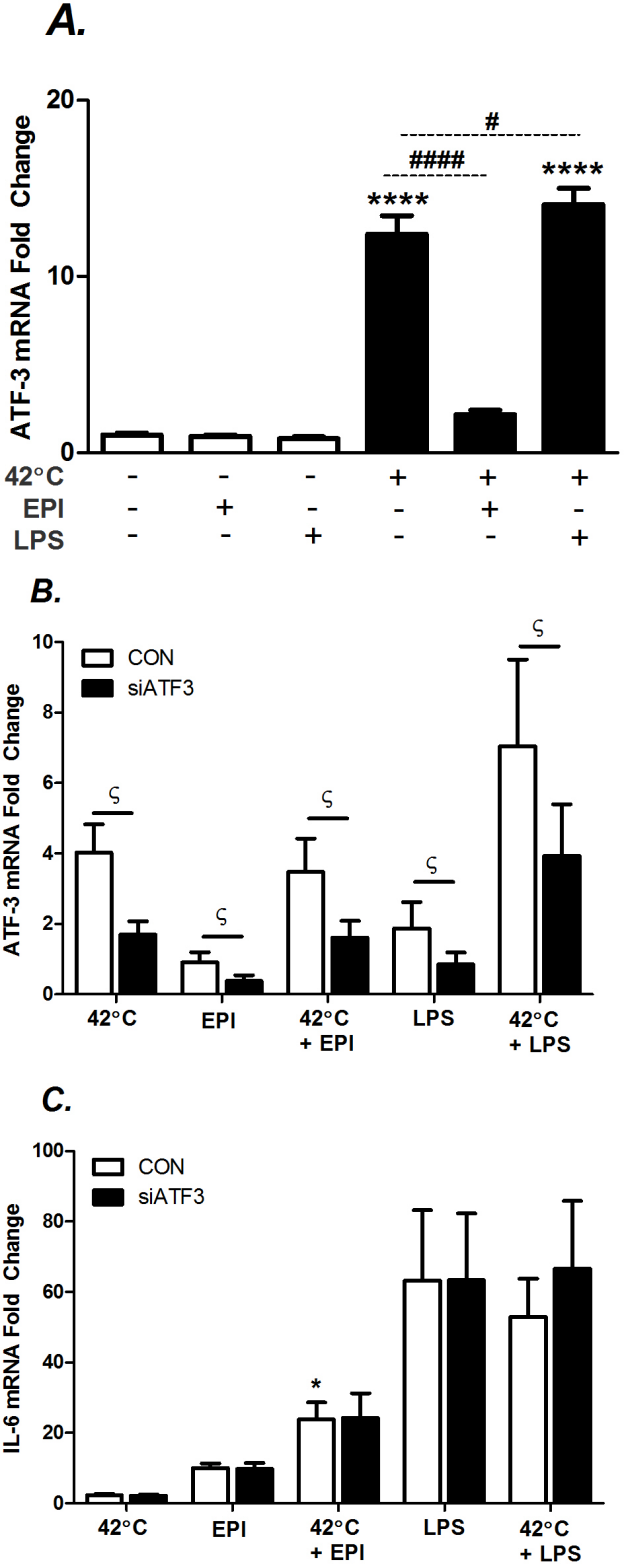
Effects of ATF-3 on IL-6 regulation in heat shocked myotubes. A) Myotubes were maintained at 37°C 3 h or 42°C 1 h and 37°C 2 h. Select cultures were co-stimulated with EPI (100 ng/ml) or LPS (1 μg/ml). B and C) Myoblasts were transfected with siATF-3 or with the control (CON), scrambled nucleotides. Next, myoblasts subsequently underwent the same treatment described in A. Cells were harvested and mRNA expression was determined by real-time qRT-PCR. Results from multiple cultures, three independent experiments, two-to-three samples per experiment; statistical tests were post-ANOVA least squares contrasts between means. * Comparison during matching experimental treatments 37 vs. 42°C; ^#^ comparison vs. 42°C; ^ϛ^ comparison vs. mismatched scramble (CON); * ^ϛ^ (P<0.05), ** (P<0.01) and **** or ^####^ (P<0.0001).

We then tested for possible altered regulation of ATF-3 in myotubes as a mechanism. In MEFs and MΦ, ATF-3 and HSF-1 interactions are required for hyperthermia-induced suppression of the IL-6 gene [31]. We first measured the response of ATF-3 mRNA to all treatment conditions and found it was upregulated, as expected, by hyperthermia alone and also elevated by LPS and hyperthermia (Fig 8A). Interestingly, ATF-3 mRNA upregulation by heat was inhibited during EPI stimulation in the heat (Fig 8A), and there was no elevation in ATF-3 by LPS or EPI treatment alone. We then tested the influence of ATF-3 responses on IL-6 mRNA by transfecting C2C12 myoblasts with ATF-3 siRNA, overnight. This successfully knocked down ATF-3 by ≈60% in all conditions (Fig 8B). However, ATF-3 knockdown had no impact on IL-6 mRNA expression during any of the treatment conditions, measured at the 3 h time point (Fig 8C).

### Effects of hyperthermia on EPI- and LPS-stimulated IL-6 mRNA and protein secretion

**Fig 9:**
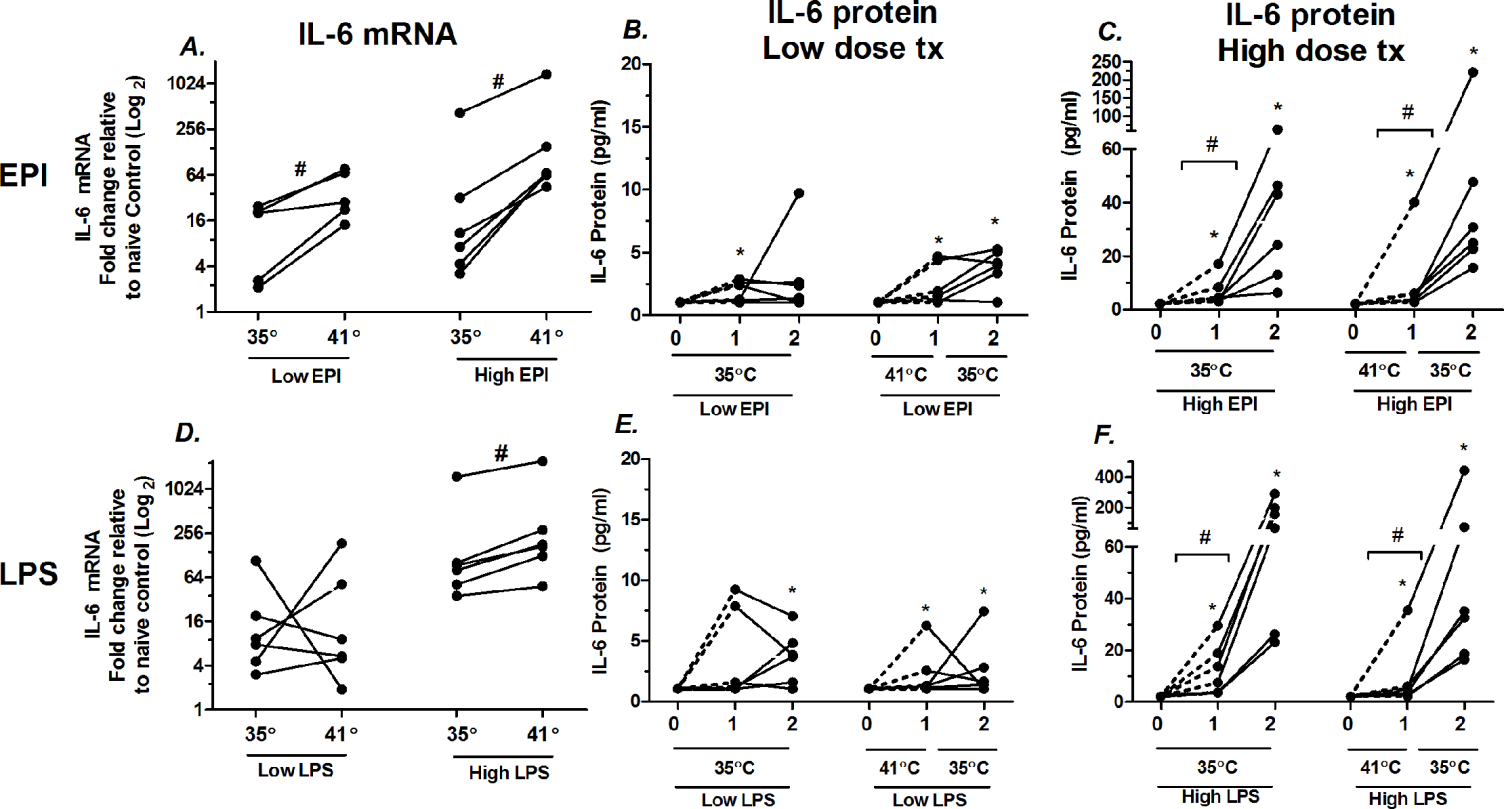
Effects of hyperthermia on low and high dose EPI- and LPS-stimulated IL-6 regulation in isolated soleus muscle. **A)** Effects of 1 h hyperthermia on IL-6 mRNA in low (1.2 ng/ml) and high dose (1 μg/ml) epinephrine (EPI). Each soleus measurement paired by animal, from one leg at 35°C; from the other leg treated for 1 h at 41°and then 1 h at 35°C. **B**. IL-6 protein secreted into the bath with exposure to low EPI, samples pulled and measured at 1 and 2 h after EPI and compared to fresh buffer (time 0). **C)** Identical to ‘B’ except high dose EPI. Bars represent paired comparisons. **D)** IL-6 mRNA response to low and high doses of LPS. **E)** IL-6 protein secretion in response to low dose LPS. **F)** IL-6 secretion in response to high dose LPS. n = 6 for all groups, except low dose mRNA to EPI (n = 5). # = P < 0.05 for paired measurements Wilcoxon signed ranks for paired samples. * = P < 0.05 for unpaired samples, Wilcoxon signed ranks for significance from lower limit threshold of IL-6 detection.

To test whether heat potentiates IL-6 mRNA in EPI- or LPS-stimulated intact muscles, soleus muscles were isolated and studied *ex vivo* in both high and low dose exposures in miniature tissue baths (Fig 9). Heat in combination with low dose EPI elevated IL-6 mRNA to a median increase of 3.6 fold (Fig 9A). Heat, in combination with high dose EPI elevated IL-6 mRNA to a median increase ≈7.0 fold. In low dose EPI, (Fig 9B) there was some scattered evidence of modest IL-6 secretion into the bath, above the Luminex threshold for detection, but there were no effects of heat exposure that could be identified in this time frame. The window of observation was limited to two hours because of previous experiments showing decay in muscle function beyond 2 h. In high dose EPI (Fig 9C) there were marked elevations in IL-6 secretion into the bath and there were significant differences in the rates of secretion between hour 1 and hour 2. However, there were no significant effects of heat + EPI vs. EPI alone on IL-6 secretion.

With LPS exposure, at low dose, there was no apparent effects of heat treatment on IL-6 mRNA expression (Fig 9D). However, in high dose LPS, 1 h of hyperthermia significantly elevated IL-6 mRNA to a median of 2.0 fold above the matched soleus samples. In low dose LPS treatment, there was no significant influence of heat treatment on protein secretion, but there was evidence of modest secretion into the bath, independent of heat. In high dose LPS, secretion into the bath was greatly elevated at both 1 and 2 h time points. There were also significant elevations in secretion between the first hour interval vs. the second hour. There were no apparent effects of heat on secretion rates within this time frame. The median rates of IL-6 secretion into the baths were substantial, reaching 29 pg/ml/h in high dose EPI treated tissues and 97 pg/ml/h in high dose LPS-treated tissues at 35°C.

ATF-3 mRNA was also evaluated in soleus after exposures to LPS, EPI and heat. There were no consistent elevations in ATF-3 induced by EPI or in low dose LPS. However, in high dose LPS, the median of ATF-3 was increased by 6.4 fold (P < 0.05, data not shown) in response to 1 h of heat treatment. This result parallels results in myoblasts shown in Fig 8A, where heat did not further elevate ATF-3 during EPI stimulation but did during LPS stimulation.

## Discussion

Our results demonstrate that hyperthermia potentiates EPI- and LPS-induced IL-6 mRNA in both isolated myotubes and intact soleus when supraphysiologic doses of these mediators are used. It also stimulates IL-6 mRNA at a physiological dose of EPI in isolated soleus. In myotubes, hyperthermia further stimulated IL-6 protein secretion during supraphysiologic EPI exposure, but either inhibited or did not change LPS-induced secretion. The results for LPS stimulation in the heat are in contrast to previous findings in fibroblasts and peritoneal macrophages exposed to the same supraphysiologic concentrations of LPS. In those experiments IL-6 mRNA was consistently inhibited by heat shock [29,31].

### Considerations regarding sensitivity of muscle to LPS and EPI

Our major outcomes for the effects of LPS are reported at supraphysiologic doses. Attempts were made to study lower doses in both myotubes and soleus that are closer to plasma values seen in severe sepsis i.e. ≈200 pg/ml [13]. However, in myotubes, significant secretion was only observed at ≥10 ng/ml, ≈50 times the values in septic patients, with no IL-6 secretion at 1 ng/ml. Isolated soleus muscles were then exposed to 200 pg/ml LPS, to test for the possibility that intact soleus might be more sensitive than myofibers. However, no increase in IL-6 mRNA or protein were observed at this concentration, with or without heat. One might conclude from this that muscle fibers are not sensitive to biologically relevant LPS. However, low sensitivities to this particular 026:B6 *e coli* LPS preparation are seen in inflammatory cells, which are well known mediators of LPS-responsiveness *in vivo*. For example, neutrophils in buffer require >100 ng/ml of 026:B6 to induce a ≈15% elevation in chemotactic responsiveness [43]. Isolated dendritic cells require ≈1 μg/ml of 026:B6 to induce ≈60% of the max IL-6 response [44]. In human monocytes, 1 ng/ml is needed to get ≈10% max cytokine response [45]; whereas 100 ng/ml of 06:B6 are needed to induce any measurable cytokine responses in whole blood [46]. Therefore, the fact that apparently pathophysiological concentrations LPS failed to stimulate IL-6 in muscle is not surprising based on responses of other phenotypes. There are several possible reasons. First, both inflammatory cells and C2C12 cells have been shown to have sensitivity levels to 026:B6 LPS that are several orders of magnitude lower compared to other forms of bacterial wall products [2,46]. How this relates to the kinds of LPS seen in the blood of human sepsis patients is uncertain. Second, in aqueous media, LPS self-organizes into micelles or pre-micelle aggregates that congregate [47] and are likely to interact with other membrane lipids, restricting diffusion into intact muscle or TLR engagement in cell culture. Aggregation is avoided *in vivo* because of the presence of LPS binding proteins, which promote toll receptor binding at low concentration [48].

A similar argument can be made for EPI experiments. For example, we based our dosing on the previous work of Frost et al. [5] in C2C12 cells. Their work shows an almost identical EC_50_ to EPI that is classically reported for isolated aortic ring contractions [49]. But these are levels much higher than are ever observed *in vivo,* to our knowledge. Nevertheless, in this study the 1.2 ng/ml EPI dose in the isolated soleus appeared to result in significant mRNA stimulation in the presence of heat, suggesting that intact soleus has sensitivity to EPI in this range. That physiologic concentrations of EPI can stimulate IL-6 in skeletal muscle is supported by the work of Frost et al. [5] who used a constant perfusion system to increase plasma EPI in rats to ≈1.4 ng/ml, maintained over 2 hours. IL-6 mRNA increased approximately 40 fold and intramuscular protein increased 15 fold. The requirement for high levels of EPI *in vitro* to induce physiological effects may reflect the fact that EPI is notoriously vulnerable to oxidation reactions in oxygenated NaCl solutions, even when made with deionized water [50]. Based on predictive oxygenation rates, the EPI in these experiments would have only lasted a few minutes, limiting effective diffusion into the muscle, and resulting in little time available for receptor engagement.

### Insights into IL-6 regulation in muscle during heat

Our data support our hypothesis that physiological hyperthermia can intensify skeletal muscle’s mRNA responses to receptor-mediated IL-6 gene expression, at least at supraphysiologic concentrations. We also found that both HSF-1 and the AP-1 element on the IL-6 promoter are essential for these interactions. Interestingly, a reduction of endogenous HSF-1 (d.n.HSF-1 in Fig 3B and 3C), even during normothermic conditions, reduces EPI- and LPS-induced IL-6 promoter activity. This finding agrees with the work of Inouye et al. who showed that constitutive HSF-1 binding to the IL-6 promoter opens the chromatin structure for the maximal induction of IL-6 by LPS in MEFs [29]. In contrast, although additional expression of HSF-1 increased LPS-induced IL-6 promoter activity, it had no effect on EPI-stimulated activity (Fig 3A). Together, these results suggest that HSF-1 activation induced by heat shock is unlikely to be the “master modulator” of the effects of heat on IL-6 gene expression. However, it does appear to play an important role during LPS stimulation.

The effects of mutating the AP-1 element consistently diminished the influence of hyperthermia-induced IL-6 promoter activity during both EPI and LPS stimulation, suggesting that this pathway is critical for all interactions (Fig 4). Unexpectedly, there was no influence of the AP-1 element on LPS- or EPI-induced expression during baseline conditions. This observation is not aligned with experiments by Frost and Lang [5,42] who showed that pharmacological inhibition of JNK and p38 MAPK inhibited both EPI- and LPS-induced IL-6 production in C2C12 myoblasts. JNK engages the AP-1 promoter indirectly through activation and dimerization of the downstream c-Jun, c-Fos family of proteins. However, JNK can also phosphorylate other transcription factors such as those of the C/EBP and NF-κB families [51–53]. It is possible that differences from this previous work may reflect, a) interactions of JNK/p38 and their downstream targets with other elements of the IL-6 promoter besides the AP-1 site, b) nonspecific effects of pharmacological JNK/p38 blockers, or c) the fact that previous studies were largely performed on immature C2C12 myoblasts [5,42]. We have reported that that myoblasts and myotubes respond differently to IL-6 stimuli [17].

Heat shock affects multiple levels of cell regulation. At temperatures >40.5°C in mammals, translational arrest of nonessential proteins (non-HSPs) is expected due to inhibition of ribosomal initiation factors [54]. In fact, the ability of muscle fibers to continue to synthesize and secrete any IL-6 during heat shock is surprising, making IL-6 synthesis and secretion a heat-shock resistant response in muscle. This is particularly evident for hyperthermia alone or for combinations of hyperthermia and EPI (Fig 5). However, LPS stimulation is different in that protein secretion is often suppressed by hyperthermia, even though IL-6 mRNA and promoter activity are elevated (Figs 1 and 2). Therefore, there must be some level of ongoing inhibition of protein synthesis in hyperthermia during LPS stimulation that is unique and not evident during EPI-stimulation. The causes of this difference are unknown, but one explanation may be that selective production of heat-shock proteins could preserve the availability or activity of other proteins involved in regulation. An example of such interactions is the observation that HSP72 can interfere with IκB degradation and inhibit NF-κB signaling, a critical component of IL-6 regulation in response to LPS [55].

An alternative hypothesis that we considered is that EPI may offer a protective effect on protein synthesis. Protein synthesis is primarily controlled at the level of translation initiation [56]. Translation initiation is mediated by eukaryotic initiation factors (eIF). β-adrenergic receptor agonists recruit extracellular receptor kinase (ERK) and mammalian target of rapamycin (mTOR) to facilitate long-term potentiation [57]. We tested if EPI treatment might salvage LPS stimulated protein secretion during heat shock by superimposing EPI with LPS. However, co-stimulation with heat inhibited IL-6 mRNA (Fig 6C) and protein secretion (Fig 6D), suggesting that any protective effect of EPI stimulation on protein synthesis, is insufficient to overcome the inhibitory effect of heat during these combined stimuli.

In MEFs and MΦs, IL-6 mRNA and protein expression have been shown to be inhibited through combined effects of HSF-1 and ATF-3 on transcription [31]. Like these phenotypes, heat shock also stimulated ATF-3 mRNA in muscle myoblasts (Fig 8A). However, in myoblasts the IL-6 gene does not seem to be as sensitive to ATF-3, because knockdown across all experimental conditions (Fig 8B) had no effect on IL-6 mRNA expression (Fig 8C). Note that myoblasts had to be used in place of myotubes because the transient effect of siRNA becomes too attenuated during the 5 days necessary for differentiation. Plasmid transfection in myotubes has not been successful in our hands. Nevertheless, the lack of an ATF-3-mediated response of the IL-6 gene may be an underlying mechanism to explain the different responses of myotubes vs. other cell types such as inflammatory cells.

We also attempted to quantify secreted IL-6 protein following treatment with EPI and LPS within the tissue bath. These data showed no significant effect of heat over and above the influence of EPI or LPS alone (Fig 9B, 9C, 9E and 9F). A limitation of the approach is the deterioration of tissues over time, particularly when exposed to elevated temperatures, which limited collection to within 2 h. This may also be an explanation for why we did not see significant differences in secreted IL-6 during heat exposure compared to cell culture experiments, where we often measured protein secretion over 6 h.

### Integrative Physiological Implications

This study has shown that skeletal muscle fibers, possibly like some other parenchymal tissues [32,33], have a uniquely different IL-6 response to supraphysiologic LPS and EPI receptor activation during hyperthermia compared to fibroblasts or inflammatory cells [28,29,31]. Why might this be important? First, skeletal muscles operate above core temperature during exercise, and therefore non-infectious elevations in temperature are common. Second, our previous work has shown that signaling pathways responsible for hyperthermia-induced IL-6 expression reflect a much broader constellation of events that arise from accumulation of damaged proteins [20]. This suggests that the interactions described here may be applicable to a variety of other stress conditions. Third, the predominant and most consistent observation was an amplification of the IL-6 mRNA responsiveness to other stimuli. Functionally, this may represent a “supply side” mechanism to support IL-6 protein synthesis during conditions of heat shock, where suppression of protein synthesis is expected [54]. Fourth, differential regulation of IL-6 in hyperthermia in parenchymal cells vs. immune cells may have implications for host defense. For example, IL-6 plays critical roles in the timing, regulation and recruitment of immune cells, both locally and globally, during conditions of fever [58,59]. The secretion of localized IL-6 can thus stimulate lymphocytes and monocytes to locally marginalize and migrate into the muscle tissue, preparing for longer control of pathogens or activation of wound healing programs (as reviewed in [60]). In contrast, it may be disadvantageous for the IL-6 response to be amplified in the plasma during fever, where unfocused IL-6 secretion could result in indiscriminate activation of inflammatory cells, untargeted migration into tissues and ineffective host defense.

## Supporting Information

S1 Fig Stability of housekeeping genes to heat, epinephrine and LPS exposure. Comparison of the stability of glyceraldehyde 3-phosphate dehydrogenase GAPDH (NM_008084) compared to 2 other housekeeping genes from the same samples, hypoxanthine-guanine phosphoribosyltransverase (HPRT, NC_000086.7) and β-actin (NC_000071.6). Results expressed in raw copy number from identical matched samples using different treatments. Epinephrine (EPI, 100 ng/ml) and lipopolysacharide (LPS, 1 μg/ml), with or without 1 hr of heat treatement at 42°C. **B)** Effects of treatment and time on stabiity of the GAPDH houekeeping gene, following 1 h of heat and 2 hours of recovery at 37°C.
(TIF)

## References

[1] Welc SS, Clanton TL. The regulation of interleukin-6 implicates skeletal muscle as an integrative stress sensor and endocrine organ. Exp Physiol. 2013;98: 359–371. 10.1113/expphysiol.2012.068189 22941979 PMC5538267

[2] Frost RA, Nystrom GJ, Lang CH. Multiple Toll-like receptor ligands induce an IL-6 transcriptional response in skeletal myocytes. Am J Physiol Regul Integr Comp Physiol. 2006;290: R773–784. 10.1152/ajpregu.00490.2005 16254126

[3] Warren GL, Hulderman T, Liston A, Simeonova PP. Toll-like and adenosine receptor expression in injured skeletal muscle. Muscle Nerve. 2011;44: 85–92. 10.1002/mus.22001 21488059

[4] van Alphen GWHM, Robinette SL, Macri FJ. The adrenergic receptors of the intraocular muscles of the cat. International Journal of Neuropharmacology. 1963;2: 259–272. 10.1016/0028-3908(63)90001-7 14119486

[5] Frost RA, Nystrom GJ, Lang CH. Epinephrine stimulates IL-6 expression in skeletal muscle and C2C12 myoblasts: role of c-Jun NH2-terminal kinase and histone deacetylase activity. Am J Physiol Endocrinol Metab. 2004;286: E809–817. 10.1152/ajpendo.00560.2003 14722032

[6] Fernández-Verdejo R, Casas M, Galgani JE, Jaimovich E, Buvinic S. Exercise Sensitizes Skeletal Muscle to Extracellular ATP for IL-6 Expression in Mice. Int J Sports Med. 2013; 10.1055/s-0033-1353147 24022572

[7] Yamaki T, Wu C-L, Gustin M, Lim J, Jackman RW, Kandarian SC. Rel A/p65 is required for cytokine-induced myotube atrophy. Am J Physiol, Cell Physiol. 2012;303: C135–142. 10.1152/ajpcell.00111.2012 22592403 PMC3404521

[8] Gentile LF, Moldawer LL. DAMPs, PAMPs, and the origins of SIRS in bacterial sepsis. Shock. 2013;39: 113–114. 10.1097/SHK.0b013e318277109c 23247128 PMC3767300

[9] Patel H, Shaw SG, Shi-Wen X, Abraham D, Baker DM, Tsui JCS. Toll-like receptors in ischaemia and its potential role in the pathophysiology of muscle damage in critical limb ischaemia. Cardiol Res Pract. 2012;2012: 121237 10.1155/2012/121237 22454775 PMC3290818

[10] Zhang Q, Raoof M, Chen Y, Sumi Y, Sursal T, Junger W, et al Circulating mitochondrial DAMPs cause inflammatory responses to injury. Nature. 2010;464: 104–107. 10.1038/nature08780 20203610 PMC2843437

[11] Milne KJ, Noble EG. Exercise-induced elevation of HSP70 is intensity dependent. J Appl Physiol. 2002;93: 561–568. 10.1152/japplphysiol.00528.2001 12133865

[12] van Wijck K, Lenaerts K, van Loon LJC, Peters WHM, Buurman WA, Dejong CHC. Exercise-induced splanchnic hypoperfusion results in gut dysfunction in healthy men. PLoS ONE. 2011;6: e22366 10.1371/journal.pone.0022366 21811592 PMC3141050

[13] Opal SM, Scannon PJ, Vincent JL, White M, Carroll SF, Palardy JE, et al Relationship between plasma levels of lipopolysaccharide (LPS) and LPS-binding protein in patients with severe sepsis and septic shock. J Infect Dis. 1999;180: 1584–1589. 10.1086/315093 10515819

[14] Hahn PY, Wang P, Tait SM, Ba ZF, Reich SS, Chaudry IH. Sustained elevation in circulating catecholamine levels during polymicrobial sepsis. Shock. 1995;4: 269–273. 8564555 10.1097/00024382-199510000-00007

[15] Steensberg A, van Hall G, Osada T, Sacchetti M, Saltin B, Klarlund Pedersen B. Production of interleukin-6 in contracting human skeletal muscles can account for the exercise-induced increase in plasma interleukin-6. J Physiol (Lond). 2000;529 Pt 1: 237–242. 11080265 10.1111/j.1469-7793.2000.00237.x PMC2270169

[16] Welc SS, Clanton TL, Dineen SM, Leon LR. Heat stroke activates a stress-induced cytokine response in skeletal muscle. J Appl Physiol. 2013; 10.1152/japplphysiol.00636.2013 23928112

[17] Welc SS, Phillips NA, Oca-Cossio J, Wallet SM, Chen DL, Clanton TL. Hyperthermia increases interleukin-6 in mouse skeletal muscle. Am J Physiol, Cell Physiol. 2012;303: C455–466. 10.1152/ajpcell.00028.2012 22673618 PMC3422986

[18] Sigala I, Zacharatos P, Toumpanakis D, Michailidou T, Noussia O, Theocharis S, et al MAPKs and NF-κB differentially regulate cytokine expression in the diaphragm in response to resistive breathing: the role of oxidative stress. Am J Physiol Regul Integr Comp Physiol. 2011;300: R1152–1162. 10.1152/ajpregu.00376.2010 21325641

[19] Steensberg A, Febbraio MA, Osada T, Schjerling P, van Hall G, Saltin B, et al Interleukin-6 production in contracting human skeletal muscle is influenced by pre-exercise muscle glycogen content. J Physiol (Lond). 2001;537: 633–639. 11731593 10.1111/j.1469-7793.2001.00633.x PMC2278951

[20] Welc SS, Judge AR, Clanton TL. Skeletal muscle interleukin-6 regulation in hyperthermia. Am J Physiol, Cell Physiol. 2013;305: C406–413. 10.1152/ajpcell.00084.2013 23636453

[21] Greiwe JS, Hickner RC, Shah SD, Cryer PE, Holloszy JO. Norepinephrine response to exercise at the same relative intensity before and after endurance exercise training. J Appl Physiol. 1999;86: 531–535. 9931187 10.1152/jappl.1999.86.2.531

[22] Bosenberg AT, Brock-Utne JG, Gaffin SL, Wells MT, Blake GT. Strenuous exercise causes systemic endotoxemia. J Appl Physiol. 1988;65: 106–108. 3403455 10.1152/jappl.1988.65.1.106

[23] Webb P. Temperatures of skin, subcutaneous tissue, muscle and core in resting men in cold, comfortable and hot conditions. Eur J Appl Physiol Occup Physiol. 1992;64: 471–476. 1612090 10.1007/BF00625070

[24] Febbraio MA, Snow RJ, Stathis CG, Hargreaves M, Carey MF. Effect of heat stress on muscle energy metabolism during exercise. J Appl Physiol. 1994;77: 2827–2831. 7896628 10.1152/jappl.1994.77.6.2827

[25] Brooks GA, Hittelman KJ, Faulkner JA, Beyer RE. Tissue temperatures and whole-animal oxygen consumption after exercise. Am J Physiol. 1971;221: 427–431. 5560291 10.1152/ajplegacy.1971.221.2.427

[26] Rhind SG, Gannon GA, Shephard RJ, Buguet A, Shek PN, Radomski MW. Cytokine induction during exertional hyperthermia is abolished by core temperature clamping: neuroendocrine regulatory mechanisms. Int J Hyperthermia. 2004;20: 503–516. 10.1080/02656730410001670651 15277023

[27] Frost RA, Nystrom GJ, Lang CH. Lipopolysaccharide regulates proinflammatory cytokine expression in mouse myoblasts and skeletal muscle. Am J Physiol Regul Integr Comp Physiol. 2002;283: R698–709. 10.1152/ajpregu.00039.2002 12185005

[28] Ostberg JR, Taylor SL, Baumann H, Repasky EA. Regulatory effects of fever-range whole-body hyperthermia on the LPS-induced acute inflammatory response. J Leukoc Biol. 2000;68: 815–820. 11129648

[29] Inouye S, Fujimoto M, Nakamura T, Takaki E, Hayashida N, Hai T, et al Heat shock transcription factor 1 opens chromatin structure of interleukin-6 promoter to facilitate binding of an activator or a repressor. J Biol Chem. 2007;282: 33210–33217. 10.1074/jbc.M704471200 17766920

[30] Hagiwara S, Iwasaka H, Matsumoto S, Noguchi T. Changes in cell culture temperature alter release of inflammatory mediators in murine macrophagic RAW264.7 cells. Inflamm Res. 2007;56: 297–303. 10.1007/s00011-007-6161-z 17659435

[31] Takii R, Inouye S, Fujimoto M, Nakamura T, Shinkawa T, Prakasam R, et al Heat shock transcription factor 1 inhibits expression of IL-6 through activating transcription factor 3. J Immunol. 2010;184: 1041–1048. 10.4049/jimmunol.0902579 20018623

[32] Parikh AA, Moon MR, Kane CD, Salzman AL, Fischer JE, Hasselgren PO. Interleukin-6 production in human intestinal epithelial cells increases in association with the heat shock response. J Surg Res. 1998;77: 40–44. 10.1006/jsre.1998.5332 9698530

[33] Wang Q, Sun X, Pritts TA, Wong HR, Hasselgren PO. Induction of the stress response increases interleukin-6 production in the intestinal mucosa of endotoxaemic mice. Clin Sci. 2000;99: 489–496. 11099391

[34] Zakharova E, Grandhi J, Wewers MD, Gavrilin MA. Mycoplasma suppression of THP-1 Cell TLR responses is corrected with antibiotics. PLoS ONE. 2010;5: e9900 10.1371/journal.pone.0009900 20360862 PMC2845629

[35] Wagstaff MJ, Smith J, Collaco-Moraes Y, de Belleroche JS, Voellmy R, Coffin RS, et al Delivery of a constitutively active form of the heat shock factor using a virus vector protects neuronal cells from thermal or ischaemic stress but not from apoptosis. Eur J Neurosci. 1998;10: 3343–3350. 9824447 10.1046/j.1460-9568.1998.00339.x

[36] Zuo J, Baler R, Dahl G, Voellmy R. Activation of the DNA-binding ability of human heat shock transcription factor 1 may involve the transition from an intramolecular to an intermolecular triple-stranded coiled-coil structure. Mol Cell Biol. 1994;14: 7557–7568. 7935471 10.1128/mcb.14.11.7557-7568.1994 PMC359292

[37] Zuo J, Rungger D, Voellmy R. Multiple layers of regulation of human heat shock transcription factor 1. Mol Cell Biol. 1995;15: 4319–4330. 7623826 10.1128/mcb.15.8.4319 PMC230671

[38] Baccam M, Woo S-Y, Vinson C, Bishop GA. CD40-mediated transcriptional regulation of the IL-6 gene in B lymphocytes: involvement of NF-kappa B, AP-1, and C/EBP. J Immunol. 2003;170: 3099–3108. 12626566 10.4049/jimmunol.170.6.3099

[39] Rhee JG, Kim TH, Levitt SH, Song CW. Changes in acidity of mouse tumor by hyperthermia. Int J Radiat Oncol Biol Phys. 1984;10: 393–399. 6706733 10.1016/0360-3016(84)90060-9

[40] Kregel KC, Overton JM, Johnson DG, Tipton CM, Seals DR. Mechanism for pressor response to nonexertional heating in the conscious rat. J Appl Physiol. 1991;71: 192–196. 1917742 10.1152/jappl.1991.71.1.192

[41] Kröpfl JM, Stelzer I, Mangge H, Pekovits K, Fuchs R, Allard N, et al Exercise-induced norepinephrine decreases circulating hematopoietic stem and progenitor cell colony-forming capacity. PLoS ONE. 2014;9: e106120 10.1371/journal.pone.0106120 25180783 PMC4152172

[42] Frost RA, Nystrom GJ, Lang CH. Lipopolysaccharide and proinflammatory cytokines stimulate interleukin-6 expression in C2C12 myoblasts: role of the Jun NH2-terminal kinase. Am J Physiol Regul Integr Comp Physiol. 2003;285: R1153–1164. 10.1152/ajpregu.00164.2003 12842862

[43] Koyama S, Sato E, Nomura H, Kubo K, Miura M, Yamashita T, et al The potential of various lipopolysaccharides to release IL-8 and G-CSF. Am J Physiol Lung Cell Mol Physiol. 2000;278: L658–666. 10749742 10.1152/ajplung.2000.278.4.L658

[44] Dodge IL, Carr MW, Cernadas M, Brenner MB. IL-6 Production by Pulmonary Dendritic Cells Impedes Th1 Immune Responses. J Immunol. 2003;170: 4457–4464. 10.4049/jimmunol.170.9.4457 12707321

[45] Flegel WA, Wölpl A, Männel DN, Northoff H. Inhibition of endotoxin-induced activation of human monocytes by human lipoproteins. Infect Immun. 1989;57: 2237–2245. 2731990 10.1128/iai.57.7.2237-2245.1989 PMC313866

[46] Blix IJS, Helgeland K, Hvattum E, Lyberg T. Lipopolysaccharide from Actinobacillus actinomycetemcomitans stimulates production of interleukin-1 β, tumor necrosis factor-α, interleukin-6 and interleukin-1 receptor antagonist in human whole blood. Journal of Periodontal Research. 1999;34: 34–40. 10.1111/j.1600-0765.1999.tb02219.x 10086884

[47] Santos NC, Silva AC, Castanho MARB, Martins-Silva J, Saldanha C. Evaluation of Lipopolysaccharide Aggregation by Light Scattering Spectroscopy. ChemBioChem. 2003;4: 96–100. 10.1002/cbic.200390020 12512082

[48] Yu B, Wright SD. Catalytic properties of lipopolysaccharide (LPS) binding protein. Transfer of LPS to soluble CD14. J Biol Chem. 1996;271: 4100–4105. 8626747 10.1074/jbc.271.8.4100

[49] Furchgott RF, Bhadrakom S. Reactions of strips of rabbit aorta to epinephrine, isopropylarterenol, sodium nitrite and other drugs. J Pharmacol Exp Ther. 1953;108: 129–143. 13062084

[50] Ryan TP, Miller DM, Aust SD. The role of metals in the enzymatic and nonenzymatic oxidation of epinephrine. J Biochem Toxicol. 1993;8: 33–39. 8492301 10.1002/jbt.2570080106

[51] Ip YT, Davis RJ. Signal transduction by the c-Jun N-terminal kinase (JNK)—from inflammation to development. Current Opinion in Cell Biology. 1998;10: 205–219. 10.1016/S0955-0674(98)80143-9 9561845

[52] Koj A. Initiation of acute phase response and synthesis of cytokines. Biochimica et Biophysica Acta (BBA)—Molecular Basis of Disease. 1996;1317: 84–94. 10.1016/S0925-4439(96)00048-8 8950192

[53] Shaulian E, Karin M. AP-1 as a regulator of cell life and death. Nat Cell Biol. 2002;4: E131–136. 10.1038/ncb0502-e131 11988758

[54] Panniers R. Translational control during heat shock. Biochimie. 1994;76: 737–747. 7893824 10.1016/0300-9084(94)90078-7

[55] Chen H, Wu Y, Zhang Y, Jin L, Luo L, Xue B, et al Hsp70 inhibits lipopolysaccharide-induced NF-kappaB activation by interacting with TRAF6 and inhibiting its ubiquitination. FEBS Lett. 2006;580: 3145–3152. 10.1016/j.febslet.2006.04.066 16697380

[56] Dever TE. Gene-specific regulation by general translation factors. Cell. 2002;108: 545–556. 11909525 10.1016/s0092-8674(02)00642-6

[57] Gelinas JN, Banko JL, Hou L, Sonenberg N, Weeber EJ, Klann E, et al ERK and mTOR signaling couple beta-adrenergic receptors to translation initiation machinery to gate induction of protein synthesis-dependent long-term potentiation. J Biol Chem. 2007;282: 27527–27535. 10.1074/jbc.M701077200 17635924

[58] Fisher DT, Vardam TD, Muhitch JB, Evans SS. Fine-tuning immune surveillance by fever-range thermal stress. Immunol Res. 2010;46: 177–188. 10.1007/s12026-009-8122-9 19760057 PMC2831163

[59] Chen Q, Fisher DT, Clancy KA, Gauguet J-MM, Wang W-C, Unger E, et al Fever-range thermal stress promotes lymphocyte trafficking across high endothelial venules via an interleukin 6 trans-signaling mechanism. Nat Immunol. 2006;7: 1299–1308. 10.1038/ni1406 17086187

[60] Jones SA. Directing transition from innate to acquired immunity: defining a role for IL-6. J Immunol. 2005;175: 3463–3468. 16148087 10.4049/jimmunol.175.6.3463

