# Supplementary figures and images for: The Impact of Hyperthermia on Receptor-Mediated Interleukin-6 Regulation in Mouse Skeletal Muscle
Source: PLoS One. 2016 Feb 12;11(2):e0148927. doi: 10.1371/journal.pone.0148927 (PMC4752463; doi:10.1371/journal.pone.0148927)

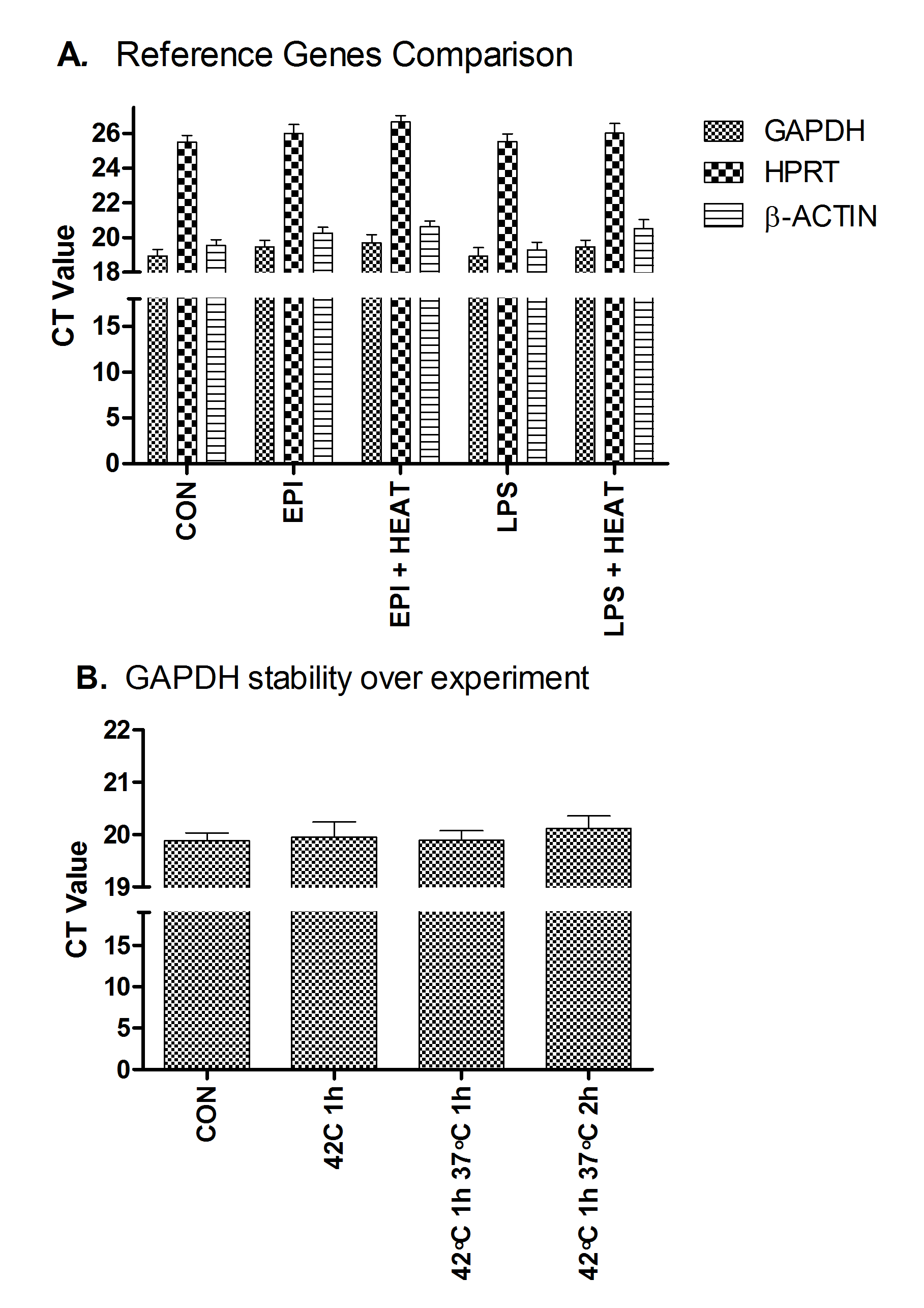

Supplement: S1 Fig — Comparison of the stability of glyceraldehyde 3-phosphate dehydrogenase GAPDH (NM_008084) compared to 2 other housekeeping genes from the same samples, hypoxanthine-guanine phosphoribosyltransverase (HPRT, NC_000086.7) and β-actin (NC_000071.6). Results expressed in raw copy number from identical matched samples using different treatments. Epinephrine (EPI, 100 ng/ml) and lipopolysacharide (LPS, 1 μg/ml), with or without 1 hr of heat treatement at 42°C. B) Effects of treatment and time on stabiity of the GAPDH houekeeping gene, following 1 h of heat and 2 hours of recovery at 37°C. (TIF) [file pone.0148927.s001.tif]
